# Supplementary material for: Overexpressing lncRNA LAIR increases grain yield and regulates neighbouring gene cluster expression in rice
Source: Nat Commun. 2018 Aug 29;9:3516. doi: 10.1038/s41467-018-05829-7 (PMC6115402; doi:10.1038/s41467-018-05829-7)
Supplement: Supplementary file 1 — Supplementary Information [file 41467_2018_5829_MOESM1_ESM.docx]

**Overexpressing lncRNA *LAIR* increases grain yield and regulates neighbouring gene cluster expression in rice**

Wang *et al.*

**
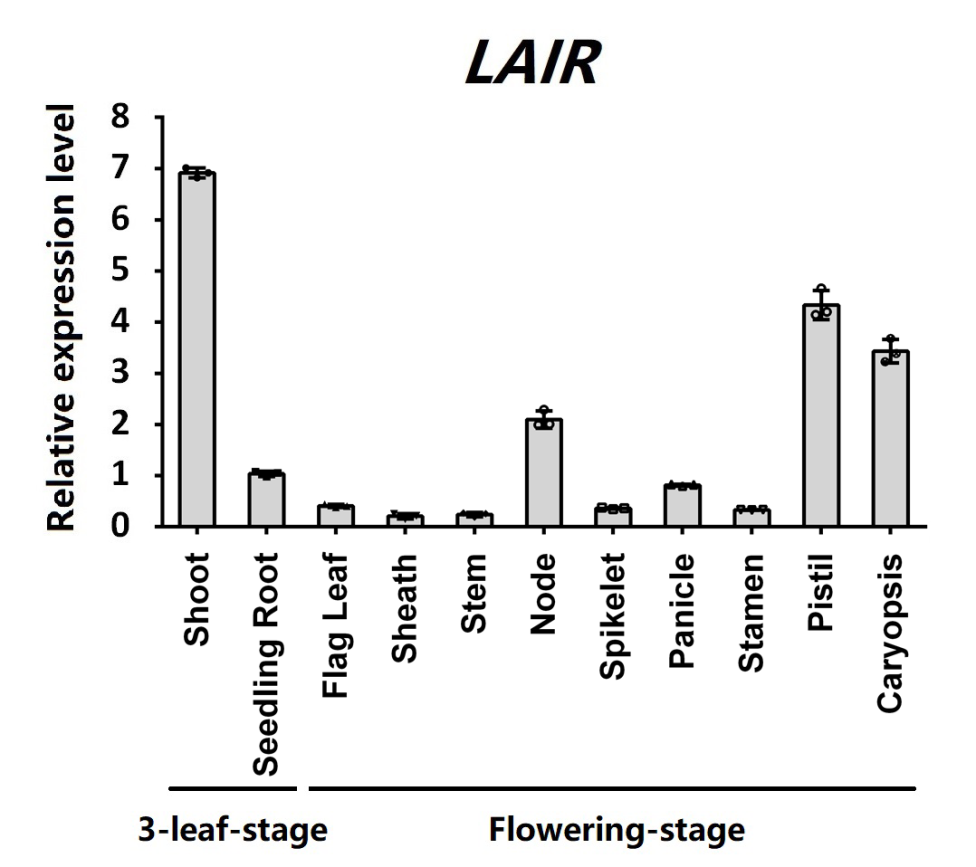
**

**Supplementary Figure 1. Analysis of *LAIR* spatial and temporal expression.** Different tissues from MH63 3-leaf-stage and flowering-stage were detected. *LAIR* showed relatively high levels in 3-leaf-stage shoot, flowering-stage node, pistil and caryopsis. Expression levels are relative to *OsActin1*. Data are presented as the mean ± standard deviation (*n* = 3).


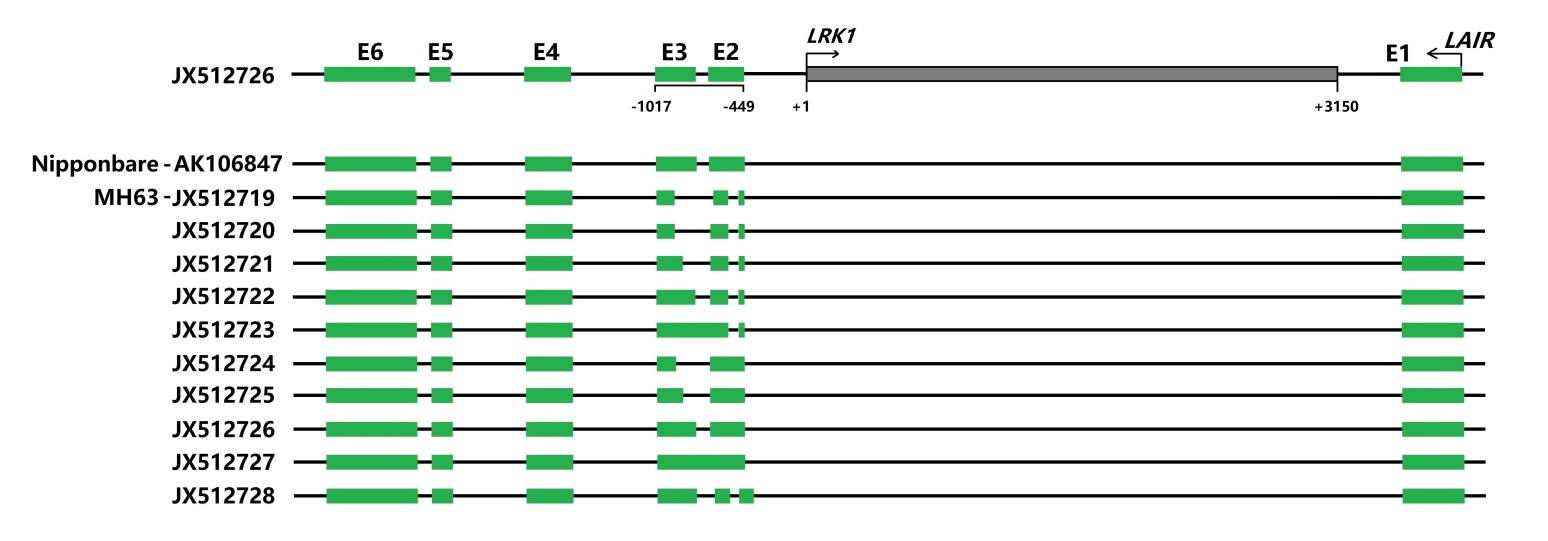


**Supplementary Figure 2. Alternatively spliced isoforms of *LAIR*.** Ten alternatively spliced isoforms of *LAIR* have been identified in the rice MH63 transcriptome. The corresponding sequences have been deposited in the GenBank database (JX512719–JX512728).


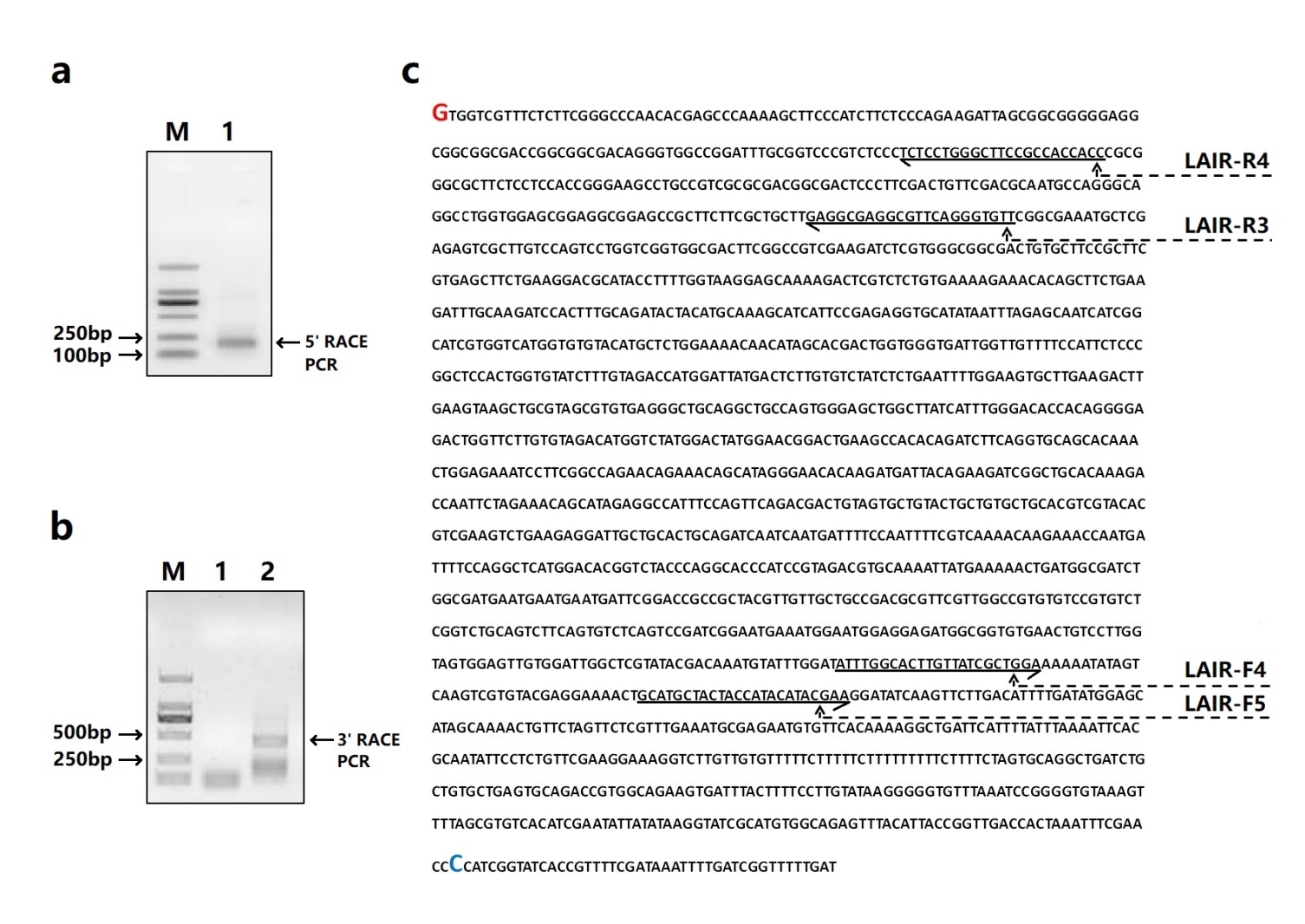


**Supplementary Figure 3. Characterization of the *LAIR* transcript.** **a.** Strand-specific amplification of *LAIR* using 5′ RACE cDNA fragments. 5′ RACE Inner Primer with Gene-Specific Primer (GSP) LAIR-R4 reverse primer [presented in (c)] was used to obtain the 5′ end products, which are presented in lane 1. Lane M: DNA marker M2000. **b.** Strand-specific amplification of *LAIR* using 3′ RACE cDNA fragments. 3′ RACE Inner Primer with GSP LAIR-F5 forward primer [presented in (c)] was used to obtain the 3′ end products, which are presented in lane 2. Lane 1: negative control lacking M-MLV (RNaseH^−^) Reverse Transcriptase; lane M: DNA marker M2000. **c.** Sequence of the predicted *LAIR*. 5′ and 3′ RACE products presented in (a) and (b) were characterized by PCR or sequencing. The transcription start site is indicated in red, while the 3′ end is indicated in blue.

**
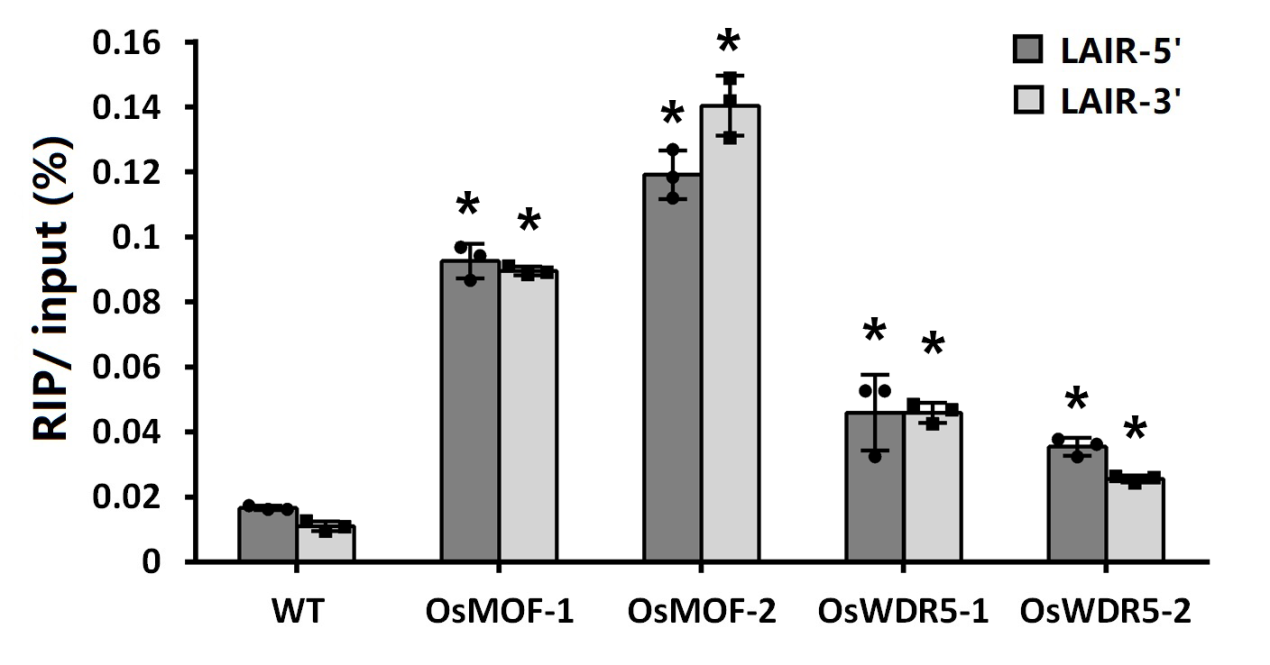
**

**Supplementary Figure 4. RIP assays of epigenetic modification-associated proteins interaction with *LAIR*.** RIP assays in rice wild-type (WT) as well as OsWDR5-FLAG (OsWDR5-1 and OsWDR5-2) and OsMOF-FLAG (OsMOF-1 and OsMOF-2) transgenic lines. The result confirmed the interaction of epigenetic modification-associated proteins with *LAIR* in Fig. 4d. The regions of LAIR-5’ and LAIR-3’ were showed in Fig. 4a. Student’s *t*-test: **P* < 0.05. Data are presented as the mean ± standard deviation (*n* = 3).

**
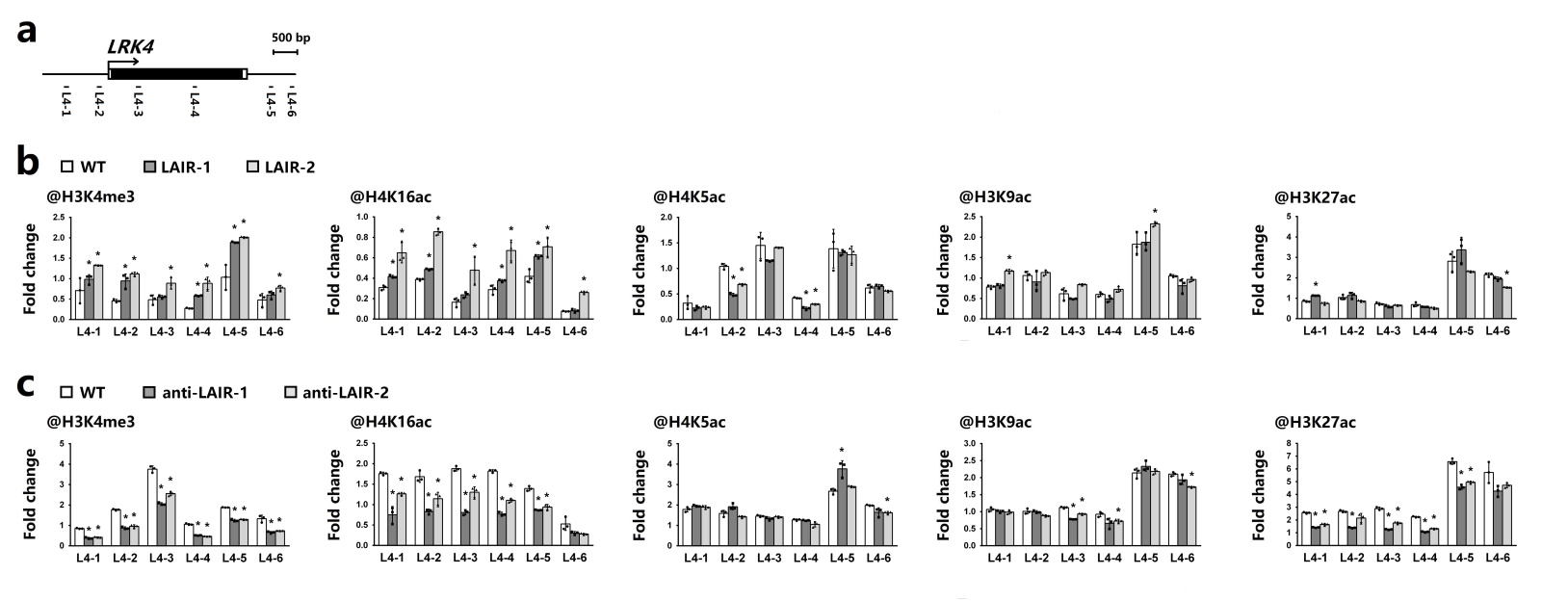
**

**Supplementary Figure 5. ChIP assays of *LRK4* locus.** **a.** Schematic representation of *LRK4* gene structure indicating the regions examined in the ChIP assays of b, c below. **b, c.** ChIP assays of *LRK4* locus. The ChIP-PCR analyses were completed for the *LRK4* chromatin regions using antibodies against H3K4me3, H4K5ac, H3K9ac, H4K16ac, and H3K27ac in rice wild-type (WT) and *LAIR*-overexpressing (LAIR-1 and LAIR-2) lines (**b**) or antisense RNAi-*LAIR* lines (anti-LAIR-1 and anti-LAIR-2) (**c**). *OsActin* was used as a reference control and for data normalization. Student’s *t*-test: **P* < 0.05. Data are presented as the mean ± standard deviation (*n* = 3).

**Supplementary Table 1. Probes used in ChIRP.**

| Gene | Probe  Name | Sequence (5’ – 3’) | even | odd |
| --- | --- | --- | --- | --- |
| *LAIR* | LAIR_1 | TGTTGGGCCCGAAGAGAAAC | | x |
|  | LAIR_2 | CGCTAATCTTCTGGGAGAAG | x |  |
|  | LAIR_3 | AGGGAGACGGGACCGCAAAT | | x |
|  | LAIR_4 | TCGAACAGTCGAAGGGAGTC | x |  |
|  | LAIR_5 | CTCAAGCAGCGAAGAAGCGG | | x |
|  | LAIR_6 | AAGCGACTCTCGAGCATTTC | x |  |
|  | LAIR_7 | GAGATCTTCGACGGCCGAAG | | x |
|  | LAIR_8 | TTCAGAAGCTCACGAAGCGG | x |  |
|  | LAIR_9 | CACAGAGACGAGTCTTTTGC | | x |
|  | LAIR_10 | GTGGATCTTGCAAATCTTCA | x |  |
|  | LAIR_11 | AATTATATGCACCTCTCGGA | | x |
|  | LAIR_12 | TACACACCATGACCACGATG | x |  |
|  | LAIR_13 | AAAACAACCAATCACCCACC | | x |
|  | LAIR_14 | CAAAGATACACCAGTGGAGC | x |  |
|  | LAIR_15 | ACACGCTACGCAGCTTACTT | | x |
|  | LAIR_16 | AAATGATAAGCCAGCTCCCA | x |  |
|  | LAIR_17 | TAGTCCATAGACCATGTCTA | | x |
|  | LAIR_18 | TGAAGATCTGTGTGGCTTCA | x |  |
|  | LAIR_19 | CTATGCTGTTTCTGTTCTGG | | x |
|  | LAIR_20 | AGCCGATCTTCTGTAATCAT | x |  |
|  | LAIR_21 | TCTGAACTGGAAATGGCCTC | | x |
|  | LAIR_22 | CATTGATTGATCTGCAGTGC | x |  |
|  | LAIR_23 | CCATGAGCCTGGAAAATCAT | | x |
|  | LAIR_24 | ATTTTGCACGTCTACGGATG | x |  |
|  | LAIR_25 | GGTCCGAATCATTCATTCAT | | x |
|  | LAIR_26 | AGACCGAGACACGGACACAC | x |  |
|  | LAIR_27 | ATTCCATTTCATTCCGATCG | | x |
|  | LAIR_28 | AACTCCACTACCAAGGACAG | x |  |
|  | LAIR_29 | GTGCCAAATATCCAAATACA | | x |
|  | LAIR_30 | CTCGTACACGACTTGACTAT | x |  |
|  | LAIR_31 | AACAGTTTTGCTATGCTCCA | | x |
|  | LAIR_32 | TCAGCCTTTTGTGAACACAT | x |  |
|  | LAIR_33 | ACAGAGGAATATTGCGTGAA | | x |
|  | LAIR_34 | ACAACAAGACCTTTCCTTCG | x |  |
|  | LAIR_35 | TCAGCCTGCACTAGAAAAGA | | x |
|  | LAIR_36 | AGTAAATCACTTCTGCCACG | x |  |
|  | LAIR_37 | CTTTACACCCCGGATTTAAA | | x |
|  | LAIR_38 | ATATTCGATGTGACACGCTA | x |  |
|  | LAIR_39 | ACCGATGGGGTTCGAAATTT | | x |
|  | LAIR_40 | GAAAACGGTGATACCGATGG | x |  |
