# Supplementary material for: Overexpressing lncRNA LAIR increases grain yield and regulates neighbouring gene cluster expression in rice
Source: Nat Commun. 2018 Aug 29;9:3516. doi: 10.1038/s41467-018-05829-7 (PMC6115402; doi:10.1038/s41467-018-05829-7)
Supplement: Supplementary file 3 — Description of Additional Supplementary Files [file 41467_2018_5829_MOESM3_ESM.pdf]

Descriptions of Additional Supplementary Files

File Name: Supplementary Data 1
Description: Primers used in this work
